# Glucocorticoid Replacement for Adrenal Insufficiency and the Development of Non-Alcoholic Fatty Liver Disease

**DOI:** 10.3390/jcm12196392

**Published:** 2023-10-06

**Authors:** Gesine Meyer, Madeleine Gruendl, Irina Chifu, Stefanie Hahner, Johanna Werner, Johannes Weiß, Tina Kienitz, Marcus Quinkler, Klaus Badenhoop, Eva Herrmann, Mireen Friedrich-Rust, Joerg Bojunga

**Affiliations:** 1 Division of Endocrinology, Medical Clinic 1, University Hospital, Goethe University Frankfurt, 60590 Frankfurt, Germanyjoerg.bojunga@kgu.de (J.B.); 2 Endocrinology and Diabetes Unit, Department of Medicine I, University Hospital Wuerzburg, 97080 Wuerzburg, Germany; 3 Division of Hepatology, Department of Medicine II, University Hospital Wuerzburg, 97080 Wuerzburg, Germany; 4 Endocrinology in Charlottenburg, 10627 Berlin, Germany; 5 Institut for Biostatistics and Mathematic Modelling, Goethe University Frankfurt, 60590 Frankfurt, Germany; 6 Division of Hepatology, Medical Clinic 1, University Hospital, Goethe University Frankfurt, 60590 Frankfurt, Germany

**Keywords:** adrenal insufficiency, glucocorticoid replacement, non-alcoholic fatty liver disease, elastography, hepatic steatosis, hepatic fibrosis, cardiometabolic risk

## Abstract

Glucocorticoid excess is a known risk factor for non-alcoholic fatty liver disease (NAFLD). Our objective was to analyse the impact of glucocorticoid replacement therapy on the development of NAFLD and NAFLD-related fibrosis and, therefore, on cardiovascular as well as hepatic morbidity in patients with adrenal insufficiency. Two hundred and fifteen individuals with primary (*n* = 111) or secondary (*n* = 104) adrenal insufficiency were investigated for hepatic steatosis and fibrosis using the fatty liver index (FLI), NAFLD fibrosis score (NAFLD-FS), Fibrosis-4 Index (FiB-4) plus sonographic transient elastography. Results were correlated with glucocorticoid doses and cardiometabolic risk parameters. The median dose of hydrocortisone equivalent was 20 mg daily, with a median therapy duration of 15 years. The presence and grade of hepatic steatosis and fibrosis were significantly correlated with cardiometabolic risk factors. We could not find any significant correlations between single, daily or cumulative doses of glucocorticoids and the grade of liver steatosis, nor with fibrosis measured via validated sonographic techniques. In patients with adrenal insufficiency, glucocorticoid replacement within a physiological range of 15–25 mg hydrocortisone equivalent per day does not appear to pose an additional risk for the development of NAFLD, subsequent liver fibrosis, or the cardiovascular morbidity associated with these conditions.

## 1. Introduction

Non-alcoholic fatty liver disease (NAFLD) is the most common liver disease worldwide, affecting up to 30–40% of adults in Western countries [1,2]. It displays a wide range of severity and is initially caused by excess hepatocellular accumulation of fat, which is termed steatosis in its isolated form. In up to one third of patients, NAFLD proceeds to non-alcoholic steatohepatitis (NASH), characterized by inflammatory processes that result in hepatocyte injury [1]. NASH can lead to progressive fibrosis and finally cirrhosis of the liver tissue and increases the risk of hepatocellular carcinoma [3,4]. The grade of liver fibrosis is unequivocally associated with increased liver disease mortality [5]. Furthermore, NAFLD represents the hepatic manifestation of metabolic syndrome. NAFLD and other manifestations of metabolic syndrome, such as type 2 diabetes mellitus and arterial hypertension, are associated bidirectionally [6]. Even in its early stages, NAFLD is an independent predictor of future cardiovascular events, and it is associated with an increased overall mortality [7,8].

Glucocorticoids induce lipid accumulation within hepatocytes by interfering with a number of metabolic processes in adipose as well as liver tissue [9,10]. Moreover, total cortisol metabolite excretion is increased in patients with steatosis and steatohepatitis compared to healthy controls [11]. Sustained hypercortisolism can therefore play an important role in the pathogenesis of NAFLD. The prevalence of NAFLD is significantly increased in patients with Cushing’s syndrome [12,13].

Little is known about the risk for NAFLD in patients requiring glucocorticoid replacement therapy due to adrenal insufficiency (AI). In patients with primary adrenal insufficiency, an elevated cardiovascular mortality has been observed [14]. Inappropriate doses leading to excess glucocorticoid exposure as well as an unphysiological diurnal variation of serum cortisol profiles are the presumed underlying causes [15,16,17]. Recently, a certain dosage effect of hydrocortisone replacement therapy on the manifestation of NAFLD could be observed in *n* = 79 patients with secondary adrenal insufficiency [18].

Liver biopsy and histopathological evaluation is the gold standard method for diagnosing NAFLD and, in particular, grades inflammation and hepatocyte injury as well as stages of fibrosis. It is, however, expensive and invasive. Therefore, non-invasive tests and procedures are increasingly replacing biopsies. To screen patients for NAFLD, fatty liver index (FLI) was developed [19]. FLI is well validated [20] and consists of clinical and laboratory data that are routinely measured and readily available. It enables a non-invasive estimation of NAFLD and is a useful predictor of cardiovascular morbidity [21]. Risk for NAFLD-related fibrosis can be assessed using NAFLD fibrosis score (NAFLD-FS) [22] and Fibrosis-4 (FIB-4) Index [23], each similarly ascertainable from routine diagnostic parameters.

B-mode ultrasound of the liver is cost-efficient and widely available, but provides only limited sensitivity as steatosis cannot be ascertained before fatty degeneration comprises a certain amount of liver tissue. In the last years, several ultrasound-based techniques have been developed in order to better assess grade of liver steatosis as well as the stage of fibrosis. The controlled attenuation parameter (CAP) is a non-invasive technique used to quantify hepatic fat content [24]. Sonographic transient elastography (TE) is an established non-invasive method to measure and quantify stiffness of liver tissue and thereby NAFLD-related fibrosis [25].

Our objective was to analyse the impact of glucocorticoid replacement therapy on patients with AI at the onset of NAFLD and NAFLD-related fibrosis and therefore on the metabolic, cardiovascular and hepatic morbidity of patients with primary (PAI) or secondary adrenal insufficiency (SAI) using non-invasive methods of evaluation.

## 2. Materials and Methods

The study was conducted from 2018 to 2021 as a multicentre trial. Assuming a two-sided error probability alpha of 0.05, an error probability beta of 0.2 and a correlation coefficient r = 0.3, a formal sample size of *n* ≥ 84 was calculated. Patients from three German endocrine centres (Goethe University Hospital Frankfurt, University Hospital Wuerzburg and a Medical Practice for Endocrinology in Berlin) were included. All patients aged 18–99 years with documented primary or secondary adrenal insufficiency under established glucocorticoid replacement therapy for at least one year were invited to participate. Exclusion criteria were: a history of endogenous Cushing’s syndrome; preceding therapy with glucocorticoids for more than 3 months and/or within the last 12 months for other indications than AI; an average alcohol consumption of >30 g/day for men, respectively, >20 g/day for women; pre-existing chronic liver disease (viral or autoimmune hepatitis, hemochromatosis, Wilson’s disease, primary biliary cholangitis, primary sclerosing cholangitis); as well as diabetes mellitus (defined as HbA1c ≥ 6.5% and/or concomitant antidiabetic medication). The study was performed in accordance with the ethical guidelines of the Helsinki Declaration and approved by the local ethics committees (ethical approval number 343/17). Written informed consent was obtained from all participants.

Data about type and duration of AI, as well as duration, preparation, dose and distribution of glucocorticoid replacement therapy, concomitant diseases, concomitant medication and alcohol consumption were collected from medical files and by interview. In order to calculate the dose of alternative glucocorticoids such as hydrocortisone (HC), the following conversion factors were used: prednisolone 5, prednisone 4, cortisone acetate 0.8, and dexamethasone 30 (https://www.endokrinologie.net/krankheiten-glukokortikoide.php; accessed on 10 August 2023). The product of daily dose of hydrocortisone equivalent in mg and duration of glucocorticoid replacement therapy in years was calculated as a marker of cumulative glucocorticoid dose in every participant.

Height, weight, waist circumference and blood pressure were measured using calibrated devices. Blood tests for glucose, insulin, HbA1c, liver enzymes (AST, ALT, GGT), bilirubin, platelet count, albumin and serum lipids (total cholesterol, high-density lipoprotein cholesterol (HDL-C), low-density lipoprotein cholesterol (LDL-C), and triglycerides (TG)) were performed under fasting conditions.

Fatty liver index (FLI) as well as NAFLD fibrosis score (NAFLD-FS) and Fibrosis-4 (FIB-4) Index were calculated for every patient using the formulas published [19,22,23]. A FLI score < 30 indicates that NAFLD can be ruled out (negative likelihood ratio 0.2), scores ≥ 60 indicate that NAFLD is present (positive predictive value 99%) [19]. Cut-off values for significant fibrosis were defined as a NAFLD-FS > 0.676 (positive predictive value > 90%), while those for the exclusion of fibrosis were set at <−1.455 (negative predictive value > 93%) [22]. A FIB-4 score is <1.3 is defined as a low-risk constellation and a score of >3.25 as a high-risk constellation for advanced fibrosis (≥F3) [26].

**Figure 1:**
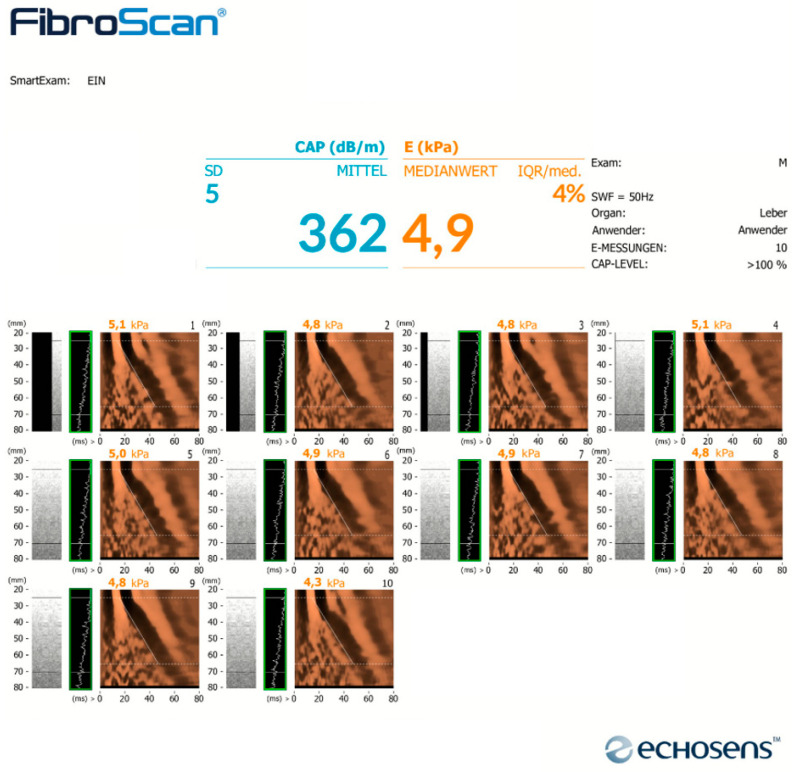
Exemplar report of sonographic transient elastography (TE) using FibroScan® including measurement of controlled attenuation parameter (CAP). Result of steatosis III°, no fibrosis.

A subgroup of participants received an ultrasound of the liver, followed by sonographic transient elastography (TE) using FibroScan^®^. TE is a sonographic method used to evaluate the elastic properties of tissues. Tissue elasticity is expressed as tensile modulus in kPa. For the classification of the stage of fibrosis, the following cut-off values were applied: for the diagnosis of significant fibrosis (F ≥ 2), 8.0 kPa; for the diagnosis of severe fibrosis (F ≥ 3), 9.0 kPa; for the diagnosis of liver cirrhosis (F = 4), 11.0 kPa [27]. Examinations were carried out under fasting conditions and during breathing baseline. Ten measurements were performed in each subject and median tensile modulus was calculated for further analyses. TE measurements were defined as reliable if the interquartile range was <30%. Controlled attenuation parameter (CAP) was assessed as part of TE. CAP enables a quantitative measurement of hepatic fat content by measuring ultrasound attenuation. Results are expressed in dB/m. The following cut-offs for the diagnosis and grading of steatosis hepatis were applied: exclusion of steatosis, <248 dB/m; steatosis I°, 248–267 dB/m; steatosis II°, 268–279 dB/m; and steatosis III° > 280 dB/m [24]. CAP and TE were adjusted for body weight, body mass index and waist circumference (Figure 1).

Clinical and laboratory characteristics were expressed as median and interquartile range and *n* (%). Since values were not normally distributed, nonparametric statistical tests were applied. Correlations between possible influencing factors (daily dose of hydrocortisone equivalent, estimated cumulative dose of hydrocortisone equivalent (daily dose in mg x duration in years), age, sex, BMI, blood pressure, waist circumference, HDL-C, LDL-C, TG, fasting glucose, HbA1c) and grade of steatosis as determined by CAP, respectively stage of fibrosis measured by TE, were calculated using Spearman’s rank correlation coefficient. For correlations between these factors and categories for NAFLD measured using FLI, respectively liver fibrosis using NAFLD-FS or FIB-4, the Jonckheere–Terpstra test was performed. A regression analysis was performed additionally for the results of TE.

In daily clinical practice, doses of glucocorticoid replacement are predominantly adjusted to patients’ physical comfort and capacity and not to body weight or surface. However, since these parameters correlate highly significantly with the risk for liver pathology, we did not include glucocorticoid doses per kg body weight nor per m^2^ body surface in our analysis to avoid a concomitant bias.

To contrast FLI, NAFLD-FS, FIB-4, CAP and TE in individuals with higher versus more physiological single doses (>15 vs. ≤15 mg HC in the morning; >5 vs. ≤5 mg HC at lunchtime; >2.5 vs. ≤2.5 mg HC in the afternoon or evening), a Wilcoxon–Mann–Whitney U-test was performed. Metabolic parameters were additionally analysed using a multiple linear regression analysis. Statistical analysis was performed using the statistical software BiAS 11.06 (Epsilon 2017, Frankfurt am Main, Germany). Statistical significance was accepted for *p* values of less than or equal to 0.05.

## 3. Results

**Figure 2:**
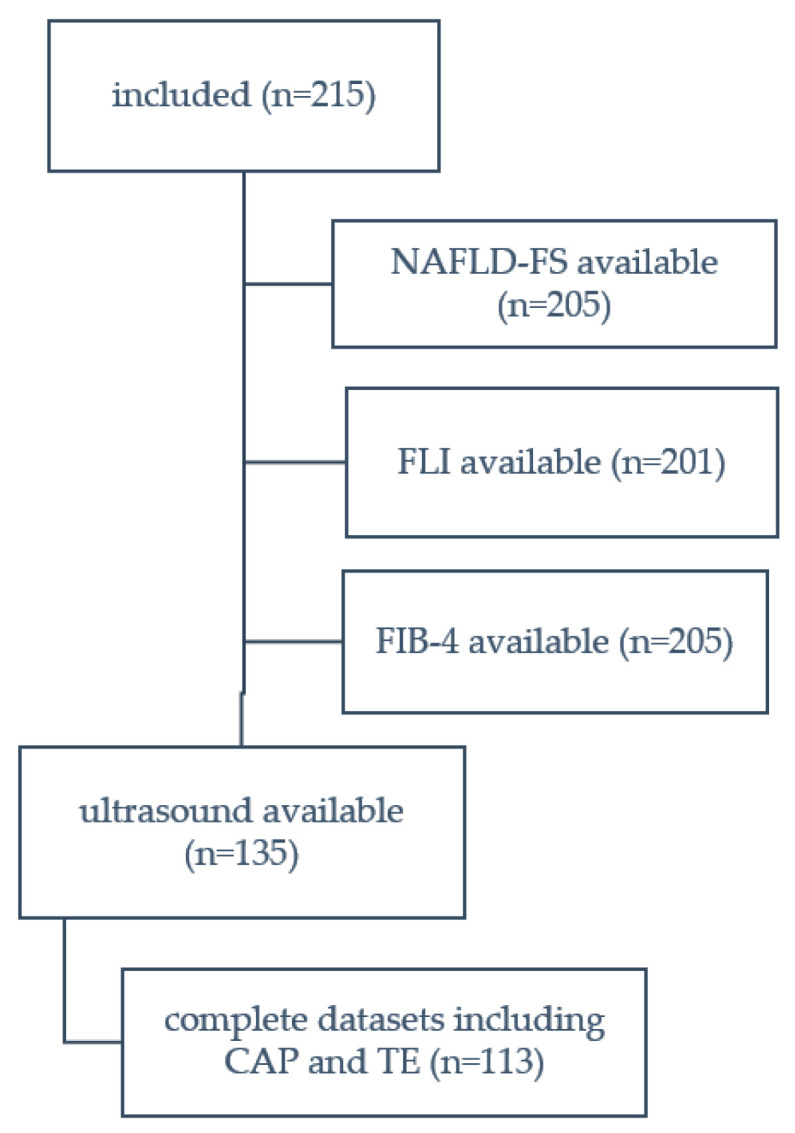
STARD diagram.

Two hundred and fifteen patients were included in our analysis, thereof 52.1% had PAI and 47.9% SAI. A complete data set, including FLI and NAFLD-FS as well as CAP and TE, was collected in *n* = 113 participants. (Figure 2).

A median dose of hydrocortisone equivalent was 20 mg daily, with a median therapy duration of 15 years. Comparable to the general population of Germany [28], about one quarter of individuals were obese with a BMI ≥ 30 kg/m^2^ (median BMI 26.4 kg/m^2^, IQR 23.7–29.7). A considerable proportion showed a waist circumference above the recommended level of 94 cm for men and 80 cm for woman. Liver enzymes (AST, ALT, GGT) were within the age- and sex-specific reference range, as were blood lipids, and there were no significant increases above the upper reference range. Patients’ characteristics are summarised in Table 1.

### 3.1. Steatosis

Steatosis could be detected in 32.3% of the participants using FLI, respectively in 43.7% by B-mode ultrasound and in 53.1% by reference using CAP (Table 2).

FLI scores and categories, B-mode ultrasound as well as CAP were significantly correlated with several cardiometabolic risk factors. See Table 3 for more details. On multiple regression analysis, only waist circumference (*p* = 0.03) remained significantly associated with CAP.).

We could not find any significant correlation between single, daily or cumulative doses of glucocorticoids and the grade of liver steatosis—nor any regarding FLI score, FLI category, B-mode ultrasound or CAP results (Table 3). Results for FLI (*p* = 0.53), B-mode ultrasound (*p* = 0.69) and CPA (*p* = 0.62) did not differ significantly between patients with PAI and those with SAI.

### 3.2. Fibrosis

Significant fibrosis was seen in 1% of the patients showing a NAFLD-FS > 0.676, while in 73.7% fibrosis could be ruled out by an NAFLD-FS < −1.455. Considering FIB-4, a high risk for an advanced fibrosis (≥F3) was found in 1.9% of patients. In 69.7% of the patients, the risk for a ≥F3 fibrosis was low. When using transient elastography, a minimum of significant fibrosis (F ≥ 2) could be detected in 7.2% of the patients (Table 4).

NAFLD-FS as well as FIB-4 scores and categories and TE were significantly correlated with several cardiometabolic risk factors. See Table 3 for more details. On multiple regression analysis for TE results, only BMI remained significantly associated with liver fibrosis measured by TE (*p* < 0.001) (Data not shown).

Regarding NAFLD-FS categories, daily glucocorticoid doses were significantly higher in individuals with a possible fibrosis than in those with no significant fibrosis (NAFLD-FS −1.455–0.676 vs. <1.455; *p* = 0.003). Furthermore, evaluating FIB-4 categories, doses of glucocorticoids were significantly higher in individuals with indeterminate condition (FIB-4 score 1.3–3.25) in comparison to those with a low risk for an advanced fibrosis (FIB-4 score < 1.3) (*p* = 0.018). Cumulative as well as individual glucocorticoid doses showed no correlation to NAFLD-FS nor FIB-4 scores or categories. Regarding the results of sonographic transient elastography (TE), we could not find any significant correlations between daily, single, or cumulative doses of glucocorticoids (Table 3). There were no significant differences between patients with PAI and those with SAI regarding NAFLD-FS (*p* = 0.66), FIB-4 (*p* = 0.64) or TE (*p* = 0.60).

In 39 patients, a modified release hydrocortisone preparation was used for replacement therapy. In comparison to patients receiving conventional hydrocortisone (*n* = 162) we could not observe any significant differences regarding liver enzymes, metabolic parameters, FLI, NAFLD-FS, and FIB-4, nor in results of B-mode ultrasound, CAP and TE. However, in the subgroup of patients receiving modified release hydrocortisone, a weak correlation could be seen between cumulative glucocorticoid dose and NAFLD-FS (rho 0.35; *p* = 0.04).

## 4. Discussion

Glucocorticoid excess is a known risk factor for hepatic as well as cardiovascular morbidity. Elevated cardiovascular risk in patients with adrenal insufficiency has been revealed to result from non-physiological glucocorticoid exposure [15,16,17,29]. Our present study provides data about the impact of glucocorticoid replacement therapy on the development of hepatic and cardiometabolic morbidity in a large, multicentre cohort comprising more than 200 individuals. To our knowledge, this is the first study using sonographic transient elastography as the non-invasive gold standard method to investigate patients receiving glucocorticoid replacement therapy for hepatic steatosis and fibrosis. So far, there are no data of comparable quality about hepatic morbidity in patients with adrenal insufficiency.

The presence and grade of hepatic steatosis as well as stage of fibrosis were significantly correlated with the well-known cardiometabolic risk factors in our cohort. No significant correlation was found between the daily nor the estimated cumulative dose of glucocorticoids and metabolic risk factors or liver enzyme levels. Glucocorticoid doses were not significantly correlated with the grade of liver steatosis measured using the fatty liver index, B-mode ultrasound and CAP. These results did not differ between patients treated with immediate release preparations and those receiving modified release hydrocortisone. In the subgroup of patients treated with modified release hydrocortisone, we noticed a weak correlation solely between the estimated cumulative glucocorticoid dose and NAFLD fibrosis score. Since several studies point to the metabolic benefits of modified release in comparison to immediate release [30,31,32,33,34]. It seems possible that patients with pre-existing metabolic comorbidity were more likely to be switched to this hydrocortisone preparation than metabolically healthy individuals and we just observed a bias. However, there were no significant differences regarding metabolic parameters between both subgroups. Our data do not indicate a lower risk for NAFLD and therefore cardiovascular morbidity for patients receiving modified release hydrocortisone.

In all patients, we found, using NAFLD-FS categories, that daily glucocorticoid doses were significantly higher in individuals with possible fibrosis than in those with no significant fibrosis, respectively, in individuals with an indeterminate risk in comparison to those with a low risk for an advanced fibrosis evaluating FIB-4 categories. Since only very few patients displayed a significant fibrosis according to score categories, a comparison with category “significant fibrosis” was not reasonable for NAFLD-FS or for FIB-4. Cumulative glucocorticoid doses showed no correlation to NAFLD-FS, FIB-4 scores or categories. Regarding the results of sonographic transient elastography (TE), which is the non-invasive gold standard method to detect liver fibrosis, we could not find any significant correlations with daily or cumulative glucocorticoid doses.

Beside an inappropriate total glucocorticoid dose, unphysiological diurnal variations of serum cortisol levels are suspected to increase the cardiometabolic risk. Single doses of 20 mg hydrocortisone in the morning and 10 mg at lunchtime were shown to generate unphysiologically high cortisol levels [29]. Therefore, we compared individuals receiving a morning dose above 15 mg, a dose at lunchtime above 10 mg or a dose in the late afternoon or evening above 2.5 mg and those receiving lower, more physiological dosages. Neither findings for steatosis (FLI, CAP) nor those for fibrosis (NAFLD-FS, FIB-4, TE) differed significantly between these two subgroups.

Previous studies pointed to an elevated cardiovascular mortality in patients with primary adrenal insufficiency. However, in these studies, data about glucocorticoid doses are missing [14] or reveal—from the present view—remarkably high daily glucocorticoid doses of up to a median of 30 mg hydrocortisone equivalent [35] in comparison to 20 mg in our cohort.

There are some limitations that have to be considered for the interpretation of our results. Our study does not permit a direct comparison between individuals taking glucocorticoid replacement therapy and those with normal adrenal function. This is based on the high incidence of NAFLD in the general population and the broad variety of factors influencing the development of NAFLD. Taking these factors and the very low incidence of adrenal insufficiency into account, compiling a sufficient and carefully matched control group is complex. Therefore, we decided to correlate the presence and grade of hepatic steatosis and the stage of fibrosis reached with the applied glucocorticoid doses, respectively, as single, daily, or cumulative doses. It is impossible to reconstruct exact cumulative life-time glucocorticoid dose due to missing traceability. Therefore, the product of the current daily dose of hydrocortisone, equivalent in mg and duration to the glucocorticoid replacement therapy in years, was used as marker for the cumulative glucocorticoid dose. This approach carries a certain risk of underestimating or overestimating the exact cumulative dose in patients who received significantly different replacement doses in the past.

In conclusion, glucocorticoid replacement therapy within a physiological range of 15–25 mg hydrocortisone equivalent per day does not appear to pose an additional risk for the development of NAFLD, subsequent liver fibrosis, or the cardiovascular morbidity associated with these conditions.

## Figures and Tables

**Table 1:** Baseline characteristics of *n* = 215 participants with adrenal insufficiency.

	Entire Group (*n* = 215)	Men (*n* = 97)	Women (*n* = 118)	PAI (*n* = 111)	SAI (*n* = 104)
Age (years)	51 (38–64)	52 (38–62)	50.5 (38–64)	44.5 (33–58.5)	55 (44–66)
Height (cm)	170 (163–176)	176.4 (172–182)	166 (160–170)	170 (163–176)	172 (164–177)
Weight (kg)	77 (67.4–89)	85 (74.7–93.6)	70.5 (63.8–80.4)	74.4 (65.1–85.6)	80 (70–92.4)
BMI (kg/m^2^)	26.4 (23.7–29.7)	27.8 (24.1–30.1)	25.8 (22.9–29.6)	25.3 (23.4–28.6)	27.6 (23.8–31.2)
Body surface area (m^2^)	1.91 (1.76–2.04)	2 (1.92–2.16)	1.8 (1.7–1.92)	1.88 (1.73–2.0)	1.93 (1.8–2.1)
Waist circumference (cm)	94 (86–104)	98 (90–107)	91 (85–99.5)	92 (85–98)	99 (87–108)
Systolic blood pressure (mmHg)	125 (115–139)	130 (120–139)	125 (113–139)	125 (115–137)	127 (118–143)
Diastolic blood pressure (mmHg)	80 (74–88)	81 (75–89)	80 (73–88)	80 (76–88)	80 (72–89)
Duration of glucocorticoid substitution (years)	15 (5–27)	15 (5–27.5)	15 (6–26)	16 (5–27.8)	14 (6–26)
Daily HC equivalent dose (mg)	20 (15–25)	20 (16.3–25)	20 (15–25)	20 (17.5–25)	20 (15–25)
HC dose in the morning (mg)	15 (10–15)	10 (10–15)	15 (10–15)	15 (10–20)	10 (10–15)
HC dose at lunchtime (mg)	5 (5–10)	5 (5–10)	5 (5–10)	5 (5–10)	5 (5–10)
HC dose in the afternoon, resp. evening (mg)	0 (0–0; range 0–10)	0 (0–0; range 0–10)	0 (0–0; range 0–10)	0 (0–0; range 0–10)	0 (0–0; range 0–10)
HbA1c (mmol/mol)	35.5 (32.2–37.7)	34.4 (33.3–37.7)	35.5 (32.2–36.6)	34.4 (32.2–37.7)	35.5 (32.2–37.7)
Insulin (pmol/L)	50 (29.2–76.4)	54.9 (31.9–97.2)	47.9 (27.1–66.0)	55.6 (29.9–90.3)	44.8 (28.5–67.4)
Fasting glucose (mmol/L)	4.94 (4.61–5.33)	4.94 (4.61–5.33)	4.88 (4.55–5.33)	5 (4.61–5.38)	4.83 (4.55–5.22)
AST (U/L)	23 (20–28)	24 (20–29)	23 (20–27)	23 (19–28)	24 (21–28)
ALT (U/L)	20 (15–28)	21.4 (15–30)	20 (14.7–26.8)	20.7 (14–29.3)	20 (15–27)
GGT (U/L)	20 (13.5–33.8)	19.8 (13.3–32.2)	20 (13.5–34.5)	20.8 (14–36.3)	19 (13–32)
Bilirubin (µmol/L)	8.6 (6.8–12.0)	8.6 (6.8–12.0)	8.6 (6.9–12.0)	8.6 (6.8–12.0)	8.6 (6.8–13.7)
Platelet count (×10^9^/L)	245 (207–286)	245 (205–287)	246 (208–281)	257 (210–319)	240 (204–270)
Albumin (g/L)	44 (42–46)	44 (42–46)	43 (41–46)	44 (42–47)	44 (41–46)
Triglycerides (mmol/L)	1.2 (0.9–1.8)	1.4 (1.0–2.1)	1.2 (0.9–1.6)	1.4 (0.9–1.9)	1.2 (1.0–1.8)
Cholesterol (mmol/L)	5.1 (4.5–5.7)	5.1 (4.4–5.7)	5.1 (4.5–5.7)	5.1 (4.6–5.9)	5.0 (4.4–5.6)
LDL-cholesterol (mmol/L)	3.0 (2.5–3.7)	3.0 (2.5–3.8)	3.0 (2.4–3.6)	3.1 (2.5–4.0)	2.8 (2.3–3.5)

Data are presented as *n* (%) or median (interquartile range). PAI: primary adrenal insufficiency; SAI: secondary adrenal insufficiency; BMI: body mass index; HC: hydrocortisone; AST: aspartate aminotransferase; ALT: alanine aminotransferase; GGT: gamma-glutamyltransferase; LDL: low-density lipoprotein.

**Table 2:** Presence and grade of hepatic steatosis.

Fatty Liver Index (FLI) (*n* = 201)		
No steatosis	FLI < 30	80 (39.8)
inconclusive	FLI 30–< 60	56 (27.9)
Steatosis	FLI ≥ 60	65 (32.3)
**Sonographic Results (*n* = 135 for B-Mode Ultrasound; *n*= 113 for CAP)**		
No steatosis	B-mode ultrasound	76 (56.3)
	CAP < 248 dB/m	53 (46.9)
Steatosis I°	B-mode ultrasound	30 (22.2)
	CAP 248–267 dB/m	14 (12.4)
Steatosis II°	B-mode ultrasound	24 (17.8)
	CAP 268–279 dB/m	3 (2.7)
Steatosis III°	B-mode ultrasound	5 (3.7)
	CAP > 280 dB/m	43 (38.1)

Data are presented as *n* (%) FLI: fatty liver index; CAP: controlled attenuation parameter.

**Table 3:** Synopsis of correlations between cardiometabolic risk factors as well as glucocorticoid doses and presence, respectively grade of hepatic steatosis or stage of fibrosis.

	Steatosis									Fibrosis								
		FLI			B-Mode			CAP			NAFLD-FS			FIB-4			TE	
	Score		Category						Score		Category		Score		Category			
age	rho 0.12 *p* = 0.103		*p* = 0.488		rho 0.26 *p* = 0.002		rho 0.18 *p* = 0.053		—		—		—		—		rho 0.11 *p* = 0.241	
weight	— †		—		rho 0.47 *p* < 0.001		rho 0.60 *p* < 0.001		—		—		rho 0.02 *p* = 0.772		*p* = 0.194		rho 0.29 *p* = 0.002	
BMI	—		—		rho 0.53 *p* < 0.001		rho 0.56 *p* < 0.001		—		—		rho 0.07 *p* = 0.325		*p* = 0.379		rho 0.24 *p* = 0.009	
systolic BP	rho 0.16 *p* = 0.019		*p* = 0.172		rho 0.30 *p* < 0.001		rho 0.18 *p* = 0.061		rho 0.28 *p* < 0.001		*p* = 0.001		rho 0.27 *p* < 0.001		*p* < 0.001		rho 0.15 *p* = 0.111	
diastolic BP	rho 0.14 *p* = 0.052		*p* = 0.060		rho 0.21 *p* = 0.013		rho −0.01 *p* = 0.893		rho 0.09 *p* = 0.216		*p* = 0.375		rho 0.05 *p* = 0.490		*p* = 0.315		rho 0.20 *p* = 0.035	
waist circumference	—		—		rho 0.54 *p* < 0.001		rho 0.65 *p* < 0.001		rho 0.31 *p* < 0.001		*p* = 0.026		rho 0.12 *p* = 0.095		*p* = 0.174		rho 0.21 *p* = 0.025	
fasting glucose	rho 0.17 *p* = 0.019		*p* < 0.001		rho −0.22 *p* = 0.012		rho −0.13 *p* = 0.176		rho 0.07 *p* = 0.316.		*p* = 0.021		rho 0.17 *p* = 0.017		*p* = 0.153.		rho −0.12 *p* = 0.232	
HbA1c	rho 0.12 *p* = 0.083		*p* < 0.001		rho −0.17 *p* = 0.052		rho 0.02 *p* = 0.849		rho 0.06 *p* = 0.362		*p* = 0.009		rho 0.25 *p* < 0.001		*p* = 0.009		rho −0.14 *p* = 0.142	
HOMA	rho 0.27 *p* < 0.001		*p* < 0.001		rho −0.04 *p* = 0.659		rho 0.11 *p* = 0.289		rho −0.12 *p* = 0.132		*p* = 0.312		rho −0.008 *p* = 0.279		*p* = 0.115		rho 0.02 *p* = 0.815	
triglycerides	—		—		rho −0.02 *p* = 0.795		eho −0.09 *p* = 0.345		rho 0.02 *p* = 0.761		*p* = 0.355		rho 0.04 *p* = 0.533		*p* = 0.477		rho −0.03 *p* = 0.775	
cholesterol	rho 0.18 *p* = 0.009		*p* = 0.125		rho −0.02 *p* = 0.861		rho −0.09 *p* = 0.365		rho 0.00 *p* = 0.993		*p* = 0.417		rho 0.11 *p* = 0.126		*p* = 0.464		rho −0.23 *p* = 0.015	
LDL	rho 0.20 *p* = 0.006		*p* = 0.011		rho −0.03 *p* = 0.689		rho −0.05 *p* = 0.593		rho 0.02 *p* = 0.825		*p* = 0.270.		rho 0.11 *p* = 0.110		*p* = 0.352		rho −0.22 *p* = 0.021	
HDL	rho −0.24 *p* = 0.019		*p* < 0.001		rho 0.07 *p* = 0.541		rho −0.01 *p* = 0.957		rho 0.03 *p* = 0.773		*p* = 0.299.		rho −0.05 *p* = 0.647		*p* = 0.197		rho 0.11 *p* = 0.438	
HC dose: daily	rho 0.10 *p* = 0.144		*p* = 0.101.		rho 0.04 *p* = 0.675		rho 0.03 *p* = 0.738		rho −0.10 *p* = 0.166		*p* = 0.003 ^1^		rho −0.18 *p* = 0.008		*p* = 0.018 ^2^		rho 0.10 *p* = 0.282	
HC dose: cumulative	rho 0.06 *p* = 0.377		*p* = 0.497		rho 0.03 *p* = 0.736		rho −0.04 *p* = 0.680		rho 0.06 *p* = 0.411		*p* = 0.151.		rho −0.03 *p* = 0.633		*p* = 0.362		rho 0.16 *p* = 0.082	
category single dose morning	*p* = 0.081		*p* = 0.377		*p* = 0.391		*p* = 0.858		*p* = 0.460		*p* = 0.642		*p* = 0.072		*p* = 0.227		*p* = 0.804	
category single dose lunch	*p* = 0.723		*p* = 0.218		*p* = 0.542		*p* = 0.079		*p* = 0.086		*p* = 0.289		*p* = 0.070		*p* = 0.396		*p* = 0.811	
category single dose evening	*p* = 0.860		*p* = 0.464		*p* = 0.849		*p* = 0.694		*p* = 0.450		*p* = 0.408		*p* = 0.371		*p* = 0.765		*p* = 0.703	

Grade of correlation of Spearman’s rank correlation coefficient marked by shades of grey (light: weak–dark: strong). † parameter is an element of the score, therefore not applicable for calculation. ^1^ no significant fibrosis (NAFLD-FS < −1.455) vs. possible fibrosis (NAFLD-FS −1.455–0.676); ^2^ low risk vs. intermediate risk for > F3 fibrosis. FLI: fatty liver index; CAP: controlled attenuation parameter; NAFLD-FS: NAFLD-Fibrosis score; FIB-4: Fibrosis-4 Index; TE: transient elastography; BMI: body mass index; BP: blood pressure; HOMA: homeostasis model assessment; LDL: low-density lipoprotein; HDL: high-density lipoprotein; HC: hydrocortisone.

**Table 4:** Presence and stage of fibrosis.

NAFLD-Fibrosis Score (NAFLD-FS) (*n* = 205)		
No significant fibrosis	NAFLD-FS < −1.455	151 (73.7)
Possible fibrosis	NAFLD-FS −1.455–0.676	52 (25.4)
Significant fibrosis	NAFLD-FS > 0.676	2 (1)
**Fibrosis-4 Index (FIB-4) (*n* = 205)**		
Low risk of ≥F3 fibrosis	FIB-4 score < 1.3	145 (69.7)
indeterminate	FIB-4 score 1.3–3.25	59 (28.4)
High risk of ≥F3 fibrosis	FIB-4 score > 3.25	4 (1.9)
**Transient elastography (TE) (*n* = 113)**		
No fibrosis	<6.7 kPa	98 (86.7)
Fibrosis stage 1 (F1)	>6.7–<8.0 kPa	7 (6.2)
Fibrosis stage 2 (F2)	>8.0–<9.0 kPa	2 (1.8)
Fibrosis stage 3 (F3)	>9.0–<11.0 kPa	3 (2.7)
Fibrosis stage 4 (F4)	>11.0 kPa	3 (2.7)

Data are presented as *n* (%) F > 2: significant fibrosis; F > 3: severe fibrosis; F = 4: liver cirrhosis.

## Data Availability

The data presented in this study are available on request from the corresponding author.
